# “The Peer Educator Is the Game-Changer of My Life”: Perceptions of Adolescents Living with HIV in DR Congo on Involving Peer Educators in the Process of HIV Disclosure

**DOI:** 10.3390/children9081239

**Published:** 2022-08-17

**Authors:** Faustin Nd. Kitetele, Gilbert M. Lelo, Cathy E. Akele, Patricia V. M. Lelo, Eric M. Mafuta, Thorkild Tylleskär, Espérance Kashala-Abotnes

**Affiliations:** 1 Department of Infectious Diseases, Kalembelembe Paediatric Hospital, Kinshasa 012, Democratic Republic of the Congo; 2 Centre for International Health (CIH), University of Bergen, 5020 Bergen, Norway; 3 Centre Neuro-Psycho-Pathologique de Kinshasa (CNPP), University of Kinshasa, Kinshasa 012, Democratic Republic of the Congo; 4 Kinshasa School of Public Health, University of Kinshasa, Kinshasa 012, Democratic Republic of the Congo

**Keywords:** HIV, disclosure, adolescent, peer approach, perception, emotional effects

## Abstract

Several approaches to the disclosure of HIV status to children and adolescents have been described. Each of these places particular emphasis on the role of parents and health care workers (HCWs) to mitigate the impact of disclosure on the adolescent without exploring the possible roles that other individuals might play in the process of disclosure. This article assesses the perceptions of adolescents living with HIV (ALHIV) about disclosure done by parents, guardians, HCWs, peer educators in the role of peer supporters, accidentally or by self-discovery, and the subsequent effects of disclosure method on their mental health. We used a qualitative study to conduct semi-structured interviews with 73 ALHIV at the Kalembelembe Paediatric Hospital, in DR Congo disclosed to by parents, guardians, HCWs, and/or peer educators, respectively, or disclosed to accidentally or by self-discovery. Microsoft Excel analysis matrix was used to organize the qualitative data. The majority of ALHIV whose disclosure involved a peer educator unanimously acknowledged the important role of the peer in accepting their HIV status, in their ART adherence, and their development of self-esteem. However, most ALHIV disclosed without involving peers declared that they had accepted their situation after a relatively long period followed by contact with the peer and integration in the self-support group. We found that the peer approach is the game-changer of the HIV status disclosure process that would allow ALHIV to accept their HIV status with minimum distress, it builds resilience, and allows them to adhere to treatment.

## 1. Introduction

Africa remains the continent most heavily affected by HIV, accounting for 68% of persons living with HIV (PLHIV) in 2021 [1]. In 2020, around 1.75 million adolescents aged 12–19 years were living with HIV, and nearly 88% of them lived in Sub-Saharan Africa (SSA) [2].

According to the Aids national program annual report 2020, the prevalence of HIV in the DRC was 1.2% in the general population. The UNAIDS DRC 2020 estimated 50,559 as the total number of adolescents living with HIV aged 10 to 19, 2118 newly infected cases, and 1794 deaths in the same age group [3].

AIDS-related deaths among adolescents have increased over the past decade while decreasing among all other age groups. Young people are at the center of the global epidemic [4].

Adolescents or youth who are living with HIV (ALHIV) may have multiple developmental, cognitive, social, contextual, and environmental challenges that impact their identification, linkage, and engagement with care [5].

As children living with HIV can grow up into adolescence and adulthood if they remain adherent to treatment and for this, they need to be informed about their situation. Disclosure of a child’s HIV status is of clinical importance in children as it will help them comply with the HIV treatment regimen [6,7]. That is why the World Health Organization (WHO) recommends that children should be informed of their HIV status before the age of 12 years [8]. Disclosure is a complex process and difficult undertaking when it involves ALHIV because it may have either negative or positive reactions [9,10].

Studies in sub-Saharan Africa (SSA) have shown that the proportion of adolescents to whom HIV disclosure has been done range from as low as 9% (South Africa) to 72% (Ouganda) [11,12,13]. This is a result of multiple factors such as parents/caregivers fear of the child disclosing his/her status to others, a lack of knowledge on how the disclosure should be made, considering that the children are young and cannot withstand the psychological impact of diagnosis [11,14,15]. It is a real challenge if we want to end the AIDS epidemic by 2030 by achieving 95% diagnosed among all PLHIV, 95% on antiretroviral therapy (ART) among diagnosed, and 95% virally suppressed among treated [16].

Several approaches to the disclosure of HIV status have been described [6]. Each of these places particular emphasis on the role of parents and health care workers (HCWs) in disclosing to children [17,18,19] without exploring the potential role that other individuals (peers,…) might play in the process of disclosure [20,21]. All the more, research shows that caregivers’ communication with children about HIV is inadequate [17].

Studies conducted in SSA and in the Democratic Republic of the Congo (DRC), have found that some ALHIV were informed of their HIV status by their primary caregiver and HCWs and suggested future studies that could explore the possible roles of other individuals might play in the process of disclosure [22,23,24]. Since 2012, a new approach has been used at the Kalembelembe Paediatric Hospital (KLPH), to improve the disclosure process. It consist of involving peer educators in all stages of the disclosure process. This approach has not been evaluated, but we have noted its adoption elsewhere in the country.

This study was designed to assess the perceptions of ALHIV about (a) different persons involved in the disclosure, whether done by involving parents, guardians, HCWs, or peer educators (PEs), and (b) different ways of disclosure, whether by a disclosure process, accidentally or by self-discovery, and (c) the induced effects. In addition, it sought to determine the linkage between different types of disclosure and anti-retroviral treatment (ART).

## 2. Subjects and Methods

### 2.1. Study Design and Setting

This was a descriptive and qualitative study using in-depth semi-structured interviews and focus group discussions conducted to elicit the ALHIVs’ perceptions about HIV disclosure and of different actors involved in the full disclosure.

The study was conducted at the Department of Infectious Diseases (DID) of KLPH located in Kinshasa, DRC with high volume of adolescents living with HIV on ART.

The KLPH is a public health facility and was in 2002 the first center in the country to provide ART and other clinical services to HIV-infected children since 2002. In 2004, KLPH through the SARA project (Sustainable AntiRetroviral Access) developed by the Kinshasa Public Health School in the DRC and at the University of North Carolina (UNC-CH) provided a family centered approach to comprehensive care and pediatric ARV treatment in the DRC.

From 2002 to 2016, an estimated 1770 HIV children and adolescents living with HIV have benefited from HIV care in this hospital.

### 2.2. Sampling

All participants were recruited among children and adolescents aged 9–19 years old and regularly follow-up at KLPH. They were recruited during regular visits or during follow-up appointments for laboratory examinations at DID of KLPH, Kinshasa, DRC. The age bracket, 8–19 years, is the extreme age of children eligible for disclosure in our center depending on the cognitive level. Although the World Health Organization (WHO) defines an adolescent as any person between ages 10 and 19, exceptionally, we disclose to pre-teens who have a sufficient degree of maturity. However, for the present study, we enrolled adolescents aged 9–19 years old.

All patients are treated and followed up using the same standard protocol at KLPH. They all benefit from peer support from day one at their enrolment in care, except for those whose parents decline. All children with parental consent will be in contact with others, benefit from psychological support, participate in psychosocial activities such as meetings, and be enrolled in the gradually HIV disclosure process established at KLPH.

All patients had access to psychological support through peer education and HCW support, and in addition, if needed with a psychologist.

Participants were recruited by purposive sampling. The participants were sampled if they were perinatally infected, were aware of their HIV status, were aged between 9 and 19 years during full disclosure, were on ART, and would have had the last viral load check completed at least 6 months before the study. Those who met the criteria for inclusion were approached through the principal investigator with the staff of DID.

In order to avoid certain biases that could be caused by the modes of HIV transmission, adolescents infected with modes, other than vertical, were excluded.

Patients who met the inclusion criteria were approached and asked to participate and received explanations on the study objectives with the consent of their parents. All parents who accepted the study signed informed consent for participation of their children, and participants older than 12 years of age provided their assent.

In total, 73 participants were recruited for the study. The sample size was determined on the basis of theoretical saturation (the point in data collection when new data no longer bring additional insights to the research questions) [25].

### 2.3. Data Collection

Face-to-face semi-structured interviews were conducted with participants. All interviews were conducted in French or Lingala (local languages), based on participant’s preferences, by principal investigator and three research assistants trained in qualitative research.

Each participant was asked to verbally respond to the questions who explored the following topics: sociodemographic characteristics of the participant, age of disclosure; quality of the person who insured the full disclosure; reasons for full disclosure, assessment of the quality of announcement, time lag between the announcement and the meeting with the PE, the feeling/effect produced after contact with PEs, the motivation that led to acceptance or non-acceptance of the HIV status, adherence to treatment and viral load within two years of disclosure. The adolescents were interviewed without their parents, to make them more at ease.

The interviews were conducted with the aid of an interview checklist including main questions and complementary questions. All interviews were recorded with notes and/or audio-recorder and then verbatim transcribed. On average the interviews lasted for 39 min (33–71 min) and proceeded until no new themes emerged.

Interviews were conducted over four months, from September to December 2018, at DID of KLPH. The viral load laboratory values were obtained from patient records charts and the KLPH’s database.

PEs’ point of view through the focus group.

The focus group comprised of nine PEs disclosed by biological parents, guardians, and HCWs with or without any preparation, and others accidentally.

PEs’ point of view through the focus group about the age and the appropriate person to ensure the full disclosure was explored.

### 2.4. Data Analysis

Data were analyzed using qualitative content analysis to identify themes and patterns related to research questions. Deductive and inductive codes were developed and applied to the data. Interviews were transcribed verbatim in Lingala, translated into French, and checked by three team members, then, combined with field notes, and mini-reports produced by the research team members after each interview and daily debriefing. The transcripts were analyzed using deductive content analysis. Data processing and analysis only focused on data related to relevant issues. The analysis was performed in three main stages. During the first stage, the transcripts were read repeatedly to become familiar with the participants’ stories and to identify themes associated with the relevant issues.

All identified themes were recorded and labelled with a unique code to compile a list of subcategories with regard to explored aspects, and an MS Excel analysis matrix was used to organize the qualitative data. The analysis matrix presented inline data from the transcript and in column associated themes/codes. Each interview transcript was represented by one line. During the second stage, the researchers used the list of subcategories to code each separate interview transcript and use the analysis matrix to organize data. During the third stage, subcategories were merged into categories corresponding to the explored aspects, and synthesis was made by seeking connections, similarities, and differences providing a means of describing these categories and generating knowledge. The process of analysis was completed by the principal investigator and discussed with other authors and local health partners. Moreover, the use of the fieldwork notes from each interview helped to assure the trustworthiness of the analysis.

### 2.5. Some Definitions

A peer educator is a member of a peer group that receives special training and information and tries to modify a person’s knowledge, attitudes, beliefs, or behaviors or among the group members [26,27,28].

A peer supporter (in behavioral health) is someone who offers emotional support on their unique lived experience with mental health conditions to provide focused support. The goals of this relationship are to generate hope, promote resilience, and strengthen the ability of another to cope with difficulty [29].

Disclosure: process involving ongoing discussions about the disease as the child or adolescent matures cognitively, socially, emotionally, and sexually [9].

Full disclosure: providing full information and knowledge about HIV [9].

Accidental discovery: telling a child or adolescent about his/her HIV status without preparing him/her and disclosing to him/her by accident or unintentionally [9]. Self-discovery: in the context of HIV, it is a self-exploration, self-reflection, or self-awareness allowing to discover his/her HIV status [30].

Self-support group (SSG): in the present study, we refer to the SSG when mentioning groups. Indeed, every adolescent that has been disclosed will automatically join the SSG to benefit from psychological support. This process of joining the SSG is done gradually and depends on the circumstances of the disclosure and the consent from the adolescent and parents.

### 2.6. Ethics Approval and Consent to Participate

The aim and nature of the study were explained to all the participants and their parents. The participants 18 years and older signed the informed consent form while those under 18 years old signed the consent form for study participation, and parental permission was also obtained. No identifying information was retained and for maintaining anonymity, the quotes were identified by gender and age.

Ethical approval for this study was obtained from the DR Congo National Health Ethics Committee at the Ministry of Health (040/CNES/BN/PMMF/2017) and the Norwegian Regional Committee for Medical and Health Research Ethics (2018/1525/REK Vest).

## 3. Results

Interviews were conducted with 73 ALWH participants, fully aware of their HIV status. Most participants in the study had been followed-up at the hospital since childhood.

All the participants participated in individual interviews and nine among them also acted as PEs in the focus group. We obtained 73 stories of disclosure from ALHIV participants.

### 3.1. Characteristics of the Participants

Forty-five (61.6%) of 73 participants were females and 52 (71.2%) had lost either one or both parents. The median age were 6.4 years (0.5–13), 14.8 years (9–19), and 20.1 years (15–24) respectively at care enrolment, during disclosure of HIV status, and the study.

### 3.2. Themes and Subthemes

Using qualitative content analysis, three themes and nine subthemes were identified (Table 1).

#### 3.2.1. Reasons for the Disclosure

For 23 of the 29 participants aged 15 years and older, the main reasons for disclosure were poor adherence to ART. The remaining six others were disclosed after following the voluntary cessation of treatment or after completing the full disclosure process put in place.

For 23 of the 44 participants younger than 15 years, had completed a full disclosure process.

Thirteen of the 44 were disclosed to due to poor adherence to ART and the remaining 8 following the child insistence, accidentally or by self-discovery.

#### 3.2.2. Who Disclosed and Where Disclosure Took Place?

A large group of participants were disclosed to by HCWs, some jointly by HCWs and PEs, some by parents or guardians, and some finally by accident, Table 2.

#### 3.2.3. Assessment of the Quality of Announcement

Most of the participants disclosed after a complete process by HCWs (26 of 32) or in association with PEs (15 of 15) reported having been satisfied with the process and the method used. One out of two self-discovery cases was also satisfied with having found the name of the disease by himself.

A 12-year-old girl said:

*“Finally, I am happy to discover my chronic illness and also to discover that I am not alone and that we are numerous. Some seniors are even in college and others had finished. Waoooh, It is fantastic.”*

A 15-year-old girl said:

*“During the process, I knew I had a chronic illness that required regular medication. By eliminating all of the chronic diseases discussed and searching the internet, I found that HIV was the only disease for which Cotrimoxazole was prescribed to prevent opportunistic infections. The next day the doctor confirmed it and I was very happy.”*

However, many of the participants disclosed to by biological parents (12 of 14), by guardians (6 of 7), and all who were accidentally disclosed said they disagreed with the way they had learned about their HIV status.

A 14-year-old girl explained:

*“During prayer, the Pastor touching my head and declare that I was completely cured of my AIDS, that I need to throw away the ARVs I was taking and I will no longer go to the hospital. People shouted and applauded from everywhere. Astonish, disappointed, crying and revolted to discover my situation.”*

A 14-year-old orphan girl said:

*“I was dead right away listening to my aunt’s husband screaming on her in the night, telling her that he did not want to see me at his house for fear of contaminating his children. The next day, my aunt came to abandon me at the hospital.”*

#### 3.2.4. Time Lag between the Announcement and the Meeting with the PE

Nearly two-thirds of ALHIV disclosed by HCWs (19 of 32), PEs and HCWs (14 of 15) had been in contact with their peers immediately or within 4 weeks after disclosure. Some of those were disclosed accidentally (1 of 3) or by self-discovery (1 of 2) had also been in touch with PEs.

On the other hand, participants who had been disclosed to by the guardians (1 of 7) or biological parents (2 of 14) had been in immediate contact with the PEs or integrated the SSG within 4 weeks after disclosure.

#### 3.2.5. The Feeling/Effect Produced after Contact with PEs

For the majority of ALHIV (64 of 73) meeting and exchanging with PEs, individually, in small groups, or during SSG had not only been a surprise but also made them realize that there were other carriers of HIV besides themselves. Moreover, it allowed them to comfort themselves, express their feelings, and see life in a positive way.

A 14-year-old boy said:

*“The meeting with the PEs was a motivation for me, a call, I had to accept my situation like them, I did not have to let myself down, I had to accept myself as such and fight to carve out a place.”*

A 14-year-old girl said:

*“I thought to be the only one and I was happy at my first meeting of SSG because I had found my real family. It has inspired me to live and to become a comfort to others.”*

An 18-year-old girl explained:

*“After the announcement, I hated my parents, for three years, we spoke by proxy. During my first meeting at SSG, I was shocked to see the enthusiasm of the ALHIV. By meeting them, I was also amazed by the relations that they had with their parents. So, one evening my conscience called me out, and I apologized to my parents for my behaviour, we cried together and today we are great friends.”*

On the other hand, a small number (9 of 73) had not been in contact with the PEs either by personal refusal or refusal from parents.

A 15-year-old girl explained:

*“I’ve been disclosed with the help of a PE, but I do not understand since I am aware, every time I see ALHIV like me in the hospital, my heart is broken and I cry. That’s why I don’t want to attend the meetings or meet the PEs.”*

A 14-year-old girl explained:

*“My teacher said that all PLHIV are impure. Why try to make me believe otherwise. Besides my aunt refuses that I meet peers or attend the SSG fearing that people think everyone at home is infected.”*

#### 3.2.6. The Motivation That Led to Acceptance or Not the HIV Status

Almost all of the ALHIV participants disclosed to by the HCWs (26 of 32) or together with a PE (14 of 15) stated that they were motivated by the preparation received, good information, advice, testimonials during SSG, support, ambitions, and the PE’s positive lifestyle.

A 15-year-old boy said:

*“When I was told, I started crying, it felt like the sky was falling on my head but when she added that she was born with it (the HIV) too, I stopped crying. I looked at her and started to listen to her attentively. She suddenly becomes interesting. As she spoke, she transformed me, she calmed me down and she finally convinced me. She became my confidant and the game-changer of my life.”*

Most of ALHIV participants disclosed to by the biological parents (9 of 14) declared that they had accepted their situation after a relatively long period, after contact with the PEs and integration into the SSG. A few participants (5 of 14) mentioned that they did not accept their situation, but tolerated it by agreeing to take the prescribed drugs.

A 13-year-old boy explained:

*“I refused to get in touch with PEs because I did not know that they were also concerned. One day I got into a meeting of SSG and I understood that all these young people that I saw and wanted to be like them were not different from me. It was a new birth for me.”*

A 17-year-old boy declared:

*“I never accepted this situation and to forget, I tried to relieve myself by using cannabis and indulge in sex. How can my parents, who are responsible for my pain, wake me up at night to advise me to take the medicine properly or I will die because I have AIDS. In front of my tears, they went to their bedroom and we did not talk more about it.”*

The majority of ALHIV disclosed by their guardians (5 of 7) reported that they were motivated by PEs, one by the grandmother, although another refused any contact with the PEs and did not accept his situation.

A 17-year-old girl explained:

*“I do not want a PE; I refuse to be cheered up because what I live in is unfair. What did I do to deserve it? I would never accept a situation that is the cause of my misfortune and the stigmatization I endure.”*

#### 3.2.7. Adherence to Treatment and Viral Load within Two Years of Disclosure

The majority of ALHIV participants responded that they had good adherence to treatment, but very few knew their last viral load. On that note, the viral load was verified together in the medical chart. Nearly three-fourths of ALHIV participants disclosed with PE involvement, two-thirds by HCWs, half by guardians and all cases of self-discovered had a suppressed viral load in the last health assessment.

#### 3.2.8. PEs’ Point of View through the Focus Group

According to their experience, and the topics retained, they told as follows:

*“The majority of biological parents disclose to children with great emotion with incomplete information. While avoiding further dialogue, they claim that the children were aware.”*

*“Some guardians take the time to discuss with ALHIV and had compassionate and empathetic and the others disclosed in total indifference.”*

*“HCWs could disclosed very well when trained, but some well-trained ones drag on in length hoping that the child will discover on its own. But others are afraid to do so and take refuge behind the parental authorization.”*

Unanimously and with all their experience, they thought that the PE was the key person in the process because he/she had undergone training, knew how to prevent and avoid certain reactions having experienced them at first hand. In addition, they are close to their audience.

Regarding the appropriate age for disclosure, they were unanimous that everything depended on the level of maturity of the child and the assimilation of information received. Nevertheless, they believed that before 12 years of age would be ideal.

## 4. Discussion

The vast majority of ALHIVs, whose disclosure involved peers, unanimously recognized the role of the latter in accepting their HIV status and that they contributed to development of self-esteem.

Adolescents naturally tend to resist any dominant source of authority such as parents and prefer to socialize more with their peers than with their families [31,32]. Research suggests that adolescents are more likely to modify their behavior and attitudes if they receive health messages from peers who face similar concerns and pressures [33,34,35].

In our study, the accompaniment of an ALHIV disclosed to by a trained peer and his/her integration into the SSG significantly mitigated the negative emotional effects generated by the disclosure. By sharing information, ideas and emotions, group participants can assist one another and find comfort in knowing that they are not alone in experiencing difficulty [36].

Meeting like-minded people is a relief and reassurance for the individual and can make him/her learn coping methods to overcome the difficulties [37,38]. By providing the necessary emotional support within an intimate environment, talking to friends and people with similar conditions can help individuals in situations where they need to be resilient [39].

Some researchers have argued that the activities carried out in peer group meetings enable participants to develop their individual frontiers and edify themselves via introspection, accepting one’s own situation, communicating with others, being concerned and responsible with respect to others, and hoping for the future. Nevertheless, these changes require the passage of time [40,41].

The presentation of useful information in the group gives rise to greater learning and a sense of overcoming obstacles. Moreover, joining a peer group and observing like-minded people make it easier to accept one’s problems and tackle them. As a result, stress decreases and boosts feelings of well-being and positive performance [42,43].

Literature on the key person or appropriate person to the disclosure seems divergent. Existing literature indicate that adolescents and their parents/caregivers prefer disclosure by caregivers with assistance from HCWs as they have the accurate knowledge of HIV [18,20,44].

However, for the vast majority of participants in our study, the key person in the disclosure was the peer educator or his/her direct involvement in all stages of the process, including the SSG.

Our study found that the ALHIV disclosed to by parents or HCWs without prior preparation or peer involvement, also those disclosed to accidentally had poor adherence or cessation of treatment with addiction, sexual abuse, and suicidal thoughts. Similar findings were reported in other previous studies [45,46,47,48]. Without psychosocial support, with limited exchanges, the ALHIV would not have dared to ask certain questions and neither the parents nor the providers would have dared approach certain subjects either. This heavy silence might have increased anxiety and depression and might have plunged the ALHIV into being resigned. Experience has shown that when people have gone through an emotional event, they want to talk about it. They thus proceed to the social passage of this experience despite the emotional reactivation provoked by this process [49]. However, when emotions such as shame or guilt have been implicated in the experience, they have an inhibitory effect on the social sharing process [50].

In our study, the disclosed and the peer share a similar situation, and the same problems, hence the inhibition of the “inhibiting effect” favoring frank dialogue, understanding, and trust. Thus, the social sharing of emotion greatly allows the emergence of demeanors that are characteristic of the relationship of attachment [51]. This socio-affective dimension of the social sharing of emotion would explain the formation of clusters articulating around peers found in our current practice. Moreover, in the role of mentor, the PE was trained to positively refine the information in order to positively improve the perceptions that would play a determining role in decision-making and sustainable behavior of the ALHIV. The latter is the socio-cognitive dimension of the social sharing of emotion. According to Rimé, social sharing oriented toward cognitive processing would promote emotional recovery [52,53,54].

The socio-affective and the socio-cognitive dimension of the peer’s involvement in the process of disclosure would largely explain the acceptance of HIV status with minimal stress, adherence to treatment, the greater retention, and suppression of the viral load of the vast majority of ALHIV disclosed. Adherence, retention to care and treatment, self-esteem, and less risk of depressive symptoms result from the dynamics of the relationship between peer and ALHIV, and psychosocial support.

What emerges from the research is that peer support roles are particularly effective, when compared to non-peer roles, in delivering on a number of elements that have been identified as central to recovery including hope, empowerment, self-management, and social inclusion [43].

Regarding the appropriate age for disclosure, PEs in the current study, similarly, to Madiba and Diko [16] in a prior study, believed that disclosure should be done when the child is mature enough to understand the implication of an HIV diagnosis; before 12 years of age would be ideal.

Although in our study, peers were at the forefront, this disclosure must always be the responsibility of the parents and HCWs [16]. It is the responsibility of HCWs to coordinate all the steps, to evaluate and identify possible depressive symptoms related to the disclosure in order to face it early.

The Finding:

We believe that the success of the peer approach in disclosure is based on the peer relationship. The dynamics of this relationship and psychosocial support would reduce the risk of recorded depressive symptoms unlike other types of disclosure.

Limitations of the study:

The number of participants is limited, particularly when dividing it into subgroups based on their experiences. More or larger studies are needed to evaluate the generalizability of our findings.

There may be bias in the responses, given the time lag between disclosure and the study. The resilience scale was not applied to measure changes brought about by the involvement of peers. The point of view of parents and HCWs was not sought to match those of the statements made by the ALHIV participants. Moreover, we did not use a rating scale to objectively assess depressive symptoms, but based on reports from ALHIV, many reported that disclosure from PEs allowed them to comfort themselves, express their feelings, and see life in a positive way.

Another limitation to the study is related to the lack of clinical outcomes of disclosure to support the impact of involving PEs in the disclosure process, as well as demonstrate clinical benefit in terms of adherence or viral loads. However, almost all ALHIV had a suppressed viral load at the last health assessment.

## 5. Conclusions

In addition to being an innovation, the involvement of peers at all stages of the HIV status disclosure process is one of the most important factors that would allow ALHIV to accept their HIV status with minimum distress. Adherence, retention to care and treatment, self-esteem, and less risk of depressive symptoms result from the dynamics of the relationship between peer and ALHIV, and psychosocial support.

The peer approach is an opportunity, at a low cost, to improve HIV status disclosure of ALHIV in resource-constrained countries.

There is a need for further studies to evaluate peer involvement, parents’ perception, and the sociocultural context, to improve the disclosure approach using PEs and better manage ALHIV.

## Figures and Tables

**Table 1:** Themes and subthemes that emerged.

Themes	Subthemes
Assessment of the process of HIV disclosure to ALHIV	- Reasons for the disclosure - Who disclosed and where disclosure took place - Assessment of the quality of announcement
Clinical and mental health impact of involving PE in the process of disclosure	- Time lag between full disclosure and the meeting with the PE - The feeling or effect produced after contact with PEs or participation in self-support group (SSG) meetings - Motivation that led to acceptance or not of the HIV status - Assessment of adherence to treatment and viral load
Perceptions of PEs about disclosure	- Appropriate age for disclosure - The key person in the process of disclosure

**Table 2:** Identity of the discloser and where disclosure took place.

	ALHIV Disclosed			Full Disclosure Location
	M	F	Total	
Health Care Workers (HCWs)	13	19	32	At the hospital
Peer educator and HCWs	4	11	15	At the hospital
Biological parents	7	7	14	At home
Guardians	2	5	7	At home
Accidental discovery:	0	1	1	At church
	0	1	1	At home
	1	0	1	At the hospital
Self-discovery	1	1	2	
TOTAL	28	45	73	

## Data Availability

The datasets generated for this study are available on request due to ethical restrictions.
